# The Association Between Generalized and Specific Problematic Internet Use and Its Gender Differences Across Different Educational Levels

**DOI:** 10.3389/fpsyg.2021.634581

**Published:** 2021-05-28

**Authors:** Yu Tian, Tengfei Zuo, Qianqian Sun, Lu Sun, Sheng Cao, Ningbo Qin

**Affiliations:** ^1^Department of Marxism, Qingdao University of Science and Technology, Qingdao, China; ^2^Shandong Research Center for Theoretical System of Socialism with Chinese Characteristics, Jinan, China

**Keywords:** GPIU, SPIU, gender difference, study period difference, cognitive–behavioral model

## Abstract

This study had two aims: to test the effect and the effect size of specific problematic Internet use (SPIU) [online shopping, online pornography, social network site (SNS) usage, and Internet gaming] on generalized problematic Internet use (GPIU) and to reveal the gender differences in GPIU and SPIU for students from the elementary school level to the university level. In total, 5,215 Chinese students (2,303 males, mean age = 16.20 years, range = 10–23 years) from four types of schools (elementary school, junior high school, senior high school, and university) provided self-report data on demographic variables (gender and educational levels), online shopping, online pornography, SNS usage, Internet gaming, and GPIU. After calculations had been controlled for demographic variables, the results indicated that (i) online shopping, online pornography, SNS usage, and Internet gaming positively predicted GPIU—and Internet gaming was the most critical predictor of GPIU—and that (ii) gender differences were revealed in Internet gaming and GPIU in all educational levels, except at senior high school where the gender differences in GPIU were not significant. Significant gender differences were found for online shopping and online pornography for all educational levels above elementary school. These results provided further understanding of the association between GPIU and SPIU and gender differences in PIU, which suggested that gender differences across different educational levels should be considered in interventions of PIU.

## Introduction

In the past decade, an increasing body of research has been conducted on problematic Internet use (PIU) in both adolescents and adults (Assunção and Matos, 2017; Lai and Kwan, 2017; Schimmenti et al., 2017). PIU can be defined as excessive or compulsive Internet usage that causes negative personal, social, and professional consequences (Davis, 2001; Han et al., 2017; Tian et al., 2017). Too much time spent on the Internet would lead individuals to suffer low level of learning engagement (Iyitoglu and Çeliköz, 2017), more negative emotions (such as severe depression and anxiety disorder) (Fayazi and Hasani, 2017; Zhao et al., 2017), and maladaptive cognitions (Tian et al., 2017), which could cause PIU. Cross-sectional studies have demonstrated that PIU is linked to several negative outcomes, including academic failure (Iyitoglu and Çeliköz, 2017), physical health difficulties (Trojak et al., 2017), psychological dysfunction (Han et al., 2017), and behavioral problems (Ivezaj et al., 2017; Quinones and Griffiths, 2017). Although a large number of studies have declared the predictors of PIU, such as personality factors, environmental factors, and cognitive factors (e.g., Davis, 2001; Sariyska et al., 2017; Tian et al., 2017), more studies are still needed to further explore the potential predictors of PIU.

## Literature Review

### Cognitive–Behavioral Model

Davis (2001) has suggested that the cognitive–behavioral model could offer theoretical explanations of the origins and pathogenesis of PIU. This model suggests that “real” cases of Internet dependence were different from artifact dependence. “Real” Internet dependence was considered to be a general, multidimensional overuse of the Internet, as part of which the user has no clear objective, defined as generalized PIU (GPIU). However, some users were dependent on a specific function of the Internet, such as online shopping, viewing online pornography, chatting in a social network site (SNS), and playing games. While these users spent a large amount of time on specific content on the Internet rather than multiple Internet functions, they also tended to be associated with symptoms of PIU, which were defined as specific PIU (SPIU). Recently, a series of studies found that individuals tended to develop GPIU by having different purposes for Internet usage (Sariyska et al., 2017; Tian et al., 2017). For example, Griffiths (2015) found that Internet gaming addiction could increase an individuals’ GPIU. Andreassen and Pallesen (2014) also found that SNS usage addiction could increase individuals’ GPIU. The results from previous studies suggest that there are significant associations between SPIU and GPIU. Therefore, examining the associations between SPIU and GPIU and identifying the important order of online shopping, online pornography, SNS usage, and Internet gaming in predicting GPIU are helpful for families and schools in developing Internet addiction interventions. Additionally, the cognitive–behavioral model has indicated that both SPIU and GPIU were different across individuals’ gender and educational levels, but only a few studies have tested them. The other aim of the present study was to test the effects of gender and educational levels on SPIU and GPIU.

### The Association Between SPIU and GPIU

Although the cognitive–behavioral model distinguishes GPIU from SPIU, the model ignores the association between GPIU and SPIU. For example, individuals who spent a large amount of time on Internet gaming also tended to have high scores on GPIU, but it does not mean that individuals with high scores on GPIU have used Internet gaming a lot: it may be that these individuals have spent the time on other Internet activities. Therefore, SPIU was significantly associated with GPIU (e.g., Mcdaniel et al., 2017). Additionally, the cognitive–behavioral model has suggested that excessive online shopping, online pornography, SNS usage, and Internet gaming could lead to symptoms of PIU (Lee and LaRose, 2007; Ballester-Arnal et al., 2016; Allen et al., 2017; Lee et al., 2017; Mcdaniel et al., 2017). However, the model did not clarify the effect size of these activities on the symptoms of PIU. For example, a previous study found that Internet gaming was more likely to be associated with symptoms of PIU rather than SNS usage (Montag et al., 2015). It may be that individuals tended to use Internet gaming to relieve the pressure or for entertainment, and the full and delightful experience of gaming could cater to their needs, leading them to spend more time on Internet gaming, even developing symptoms of PIU. In contrast, most individuals use SNS for social contact or work. Individuals tend to use it in a steady-going rather than an increasing manner, and therefore fewer individuals would suffer from PIU because of SNS usage. From this point of view, we firmly believe that the effects of online shopping, online pornography, SNS usage, and Internet gaming on GPIU were different. Evaluating the effect size of SPIU on GPIU could provide us a particular order for SPIU interventions. Additionally, evaluating the association between SPIU and GPIU is also important for the development of a cognitive–behavioral model. For example, while SPIU has a specific effect on GPIU, the path from SPIU to GPIU should be added to the model.

### Gender Differences in PIU

Gender differences in terms of PIU have been debated in literature on the topic. Some studies have indicated that male students had higher levels of PIU than female students and that female students engage more in SNS such as Facebook, Qzone, and WeChat, where they upload pictures as an act of self-expression, self-advertisement, and/or for communication/relationship maintenance (e.g., Sariyska et al., 2017; Tian et al., 2017). In contrast, male students exhibit more interest in playing computer games for entertainment (Niculović et al., 2014; Schimmenti et al., 2017). As computer games require more time spent online, male students are more likely to develop PIU. However, other studies have suggested the opposite: that female participants had higher PIU than male participants as females tend to use mobile phones more frequently (e.g., Han et al., 2017; Jiang and Zhao, 2017). Scholars have argued that females exhibit considerably higher motivation to use the Internet for interpersonal, entertainment, and shopping uses, which are positively related to PIU. These debates may be caused by either GPIU being used or one of the SPIUs being used (such as SNS usage or Internet gaming), which could just reflect the gender differences in PIU one-sidedly. Therefore, a study to test gender differences both in GPIU and SPIU simultaneously may be helpful to clarify these debates.

### The Influence of Educational Levels on Gender Differences in PIU

Educational level difference was another critical factor that could influence gender differences in PIU (Bianchi and Phillips, 2005; Baron and Campbell, 2012; Lepp et al., 2014; Piko et al., 2017). A 2-year longitudinal study revealed that female students were associated with academic-purpose computer overuse, whereas male students were associated with game-purpose computer overuse across time, which indicated gender differences in PIU (Yang et al., 2014). Furthermore, the motivations for Internet use may differ for people of different educational levels. For example, Caplan et al. (2009) revealed that “massively multiplayer online” game players tend to decrease the time they spent playing these games as their age increased. These results indicated that the motivations for Internet use could interact with an individual’s gender in predicting PIU. Therefore, identifying the interaction between gender and educational levels in predicting GPIU and SPIU could be used to further clarify the aforementioned debates.

### The Present Study

There were two aims in the present study: one was to test the effect size of SPIU (online shopping, online pornography, SNS usage, and Internet gaming) on GPIU and another was to examine the gender differences both in SPIU and GPIU for students from the elementary school level to the university level. A structural equation modeling (SEM) analysis was used to evaluate the effect size of each SPIU on GPIU, and difference tests were used to examine gender differences in SPIU and GPIU. We hypothesized that (i) each of the SPIUs tends to have a different effect size on GPIU and that (ii) the gender differences in SPIU and GPIU tend to change across different educational levels.

## Methods

### Sample and Procedure

#### Sample

In total, 5,500 students (all of them were contacted in the class) from five cities in China completed the self-reported questionnaires. Two hundred and eighty-five participants (5.18%) were excluded from the analyses due to excessive missed responses and uniform responses (missing data of participants were not included in later data analysis), resulting in a final sample of 5,215 respondents (94.82%; mean age = 16.19 years, standard deviation = 3.10). More specifically, 546 elementary school students (264 males, aged from 11 to 13 years; mean age = 11.59 years, standard deviation = 0.60), 1,710 junior school students (822 males, aged from 12 to 15 years; mean age = 13.50 years, standard deviation = 0.99), 688 senior school students (303 males, aged from 15 to 18 years; mean age = 16.22 years, standard deviation = 1.09), and 2,271 university students (914 males, aged from 17 to 21 years; mean age = 19.25 years, standard deviation = 1.74) took part in this survey; all the universities and schools were selected randomly. Additionally, all of the students’ parents were notified and given the option of refusing to allow their child’s participation. Parental consent forms were distributed to all the students. Almost 99.8% of the students’ parents (due to the young age of the children who participated) returned the consent forms, providing permission for their children’s to take part.

### Measures

#### Online Shopping

Online shopping was assessed using the Online Shopping Addiction Scale (Zhao et al., 2017). Participants answered 18 items (e.g., “I frequently think about how to gain more spare time or money to spend on online shopping”) on a seven-point scale ranging from 1 = “Completely disagree” to 7 = “Completely agree.” The Cronbach’s α coefficient for this sample was 0.96.

#### Online Pornography

The viewing of online pornography was assessed using the Online Pornography Scale (Kor et al., 2014). Participants answered 12 items (e.g., “I feel I cannot stop watching pornography online”) on a seven-point scale ranging from 1 = “Strongly disagree” to 7 = “Strongly agree.” The Cronbach’s α coefficient for this sample was 0.94.

#### SNS Usage

SNS usage was assessed using the Facebook Usage Scale (Ellison et al., 2007). In this study, the Facebook context was changed to the contexts of WeChat and Qzone, which were the most popular SNSs in China at the time of this study. Participants answered 12 items (e.g., “I feel out of touch when I haven’t logged onto WeChat/Qzone for a while”) on a seven-point scale ranging from 1 = “Strongly disagree” to 7 = “Strongly agree.” The Cronbach’s α coefficient for this sample was 0.85.

#### Internet Gaming

Internet gaming was assessed using the Internet gaming disorder test (Király et al., 2015). Participants answered 10 items (e.g., “Have you risked or lost a significant relationship because of gaming?”) on a seven-point scale ranging from 1 = “Strongly disagree” to 7 = “Strongly agree.” The Cronbach’s α coefficient for this sample was 0.92.

#### Generalized PIU

GPIU was assessed using the Problematic Internet Use Scale (Gómez et al., 2017). Participants answered 11 items (e.g., “You need to spend more and more time connected to the Internet to feel comfortable”) on a seven-point scale ranging from 1 = “Strongly disagree” to 7 = “Strongly agree.” The Cronbach’s α coefficient for the present sample was 0.88.

### Statistical Analyses

Firstly, SEM was used to test the regression coefficients of online shopping, online pornography, SNS usage, and Internet gaming on GPIU; the largest standardized regression coefficient was the most critical predictor of GPIU. Secondly, multivariate analysis of variance (MANOVA) was used to test the gender differences in online shopping, online pornography, SNS usage, Internet gaming, and GPIU across the different educational levels. SEM analyses were conducted using MPLUS 7.0 and a robust maximum likelihood estimation with microarray background correction (Ullman, 2006). In this study, χ^2^ was used as the primary criterion to evaluate the model fit (Hu and Bentler, 1999). In addition, the root mean square error of approximation (RMSEA), Tucker–Lewis index (TLI), and the comparative fit index (CFI) were also used to evaluate the model. RMSEA values of ≤0.05, ≤0.08, and ≥0.1 indicate good model fit, reasonable model fit, and poor model fit, respectively. TLI and CFI values of >0.95 but <1 indicate a good model fit (Byrne, 2012). SPSS version 21.0 was used to conduct a bivariate correlation analysis and MANOVA.

## Results

### Descriptive Statistics and Correlation Analysis

The skewness and kurtosis values for each variable are presented in Table 1. Mardia’s value for each studied variable was smaller than 3, which indicated that the data in the present study were normal. Table 2 depicts Pearson’s correlation, mean, and standard deviation values among all the observed factors in the measurement model. Educational level (1 = elementary school; 2 = junior high school; 3 = senior high school; and 4 = university) was positively associated with gender (1 = male and 2 = female), online shopping, online pornography, SNS usage, and GPIU. Gender was positively associated with online shopping, and SNS usage, whereas it was negatively associated with online pornography and Internet gaming. Moreover, online shopping, online pornography, SNS usage, Internet gaming, and GPIU were positively associated with each other.

**TABLE 1:** Skewness and kurtosis values for each variable.

**Items**	**Online shopping**		**Online pornography**		**SNS usage**		**Internet gaming**		**GPIU**	
	**Skewness**	**Kurtosis**	**Skewness**	**Kurtosis**	**Skewness**	**Kurtosis**	**Skewness**	**Kurtosis**	**Skewness**	**Kurtosis**
Elementary school	1.84	0.49	1.73	1.31	0.34	–0.72	1.42	0.24	0.87	0.61
Junior high school	1.29	1.58	2.37	0.71	0.04	–0.68	0.78	0.21	0.40	–0.08
Senior high school	0.96	0.41	2.63	0.73	–0.35	–0.41	0.96	0.34	0.23	–0.12
University	0.82	0.82	1.98	0.46	–0.57	0.41	0.86	0.46	0.34	0.07

**TABLE 2:** Mean, standard deviation, and Pearson’s correlation values of the studied variables.

**Variables**	***M* ± SD**	**1**	**2**	**3**	**4**	**5**	**6**	**7**
1. Educational level	2.90 ± 1.08	1						
2. Gender	1.55 ± 0.52	0.08**	1					
3. Online shopping	40.10 ± 20.37	0.25**	0.12**	1				
4. Online pornography	19.70 ± 12.17	0.08**	−0.19**	0.34**	1			
5. SNS usage	19.15 ± 6.00	0.30**	0.04**	0.05**	0.08**	1		
6. Internet gaming	24.45 ± 12.93	0.00	−0.31**	0.40**	0.21**	0.18**	1	
7. GPIU	37.28 ± 13.28	0.18**	–0.02	0.22**	0.43**	0.51**	0.53**	1

*Coefficient is significant at the 0.05 level. **Coefficient is significant at the 0.01 level.

### SEM Analysis for the Effect Size of SPIU on GPIU

**FIGURE 1:**
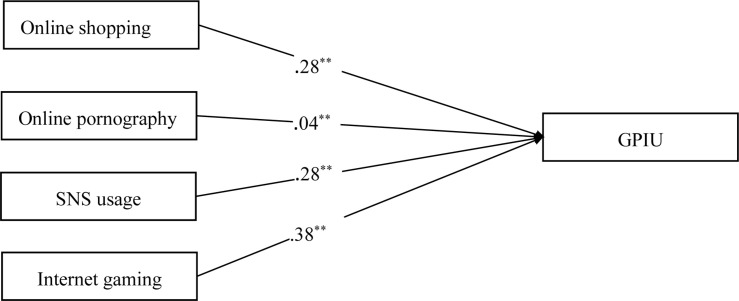
Effects of online shopping, online pornography, SNS usage, and Internet gaming on GPIU.

SEM analyses were used to test the effects of online shopping, online pornography, SNS usage, and Internet gaming on GPIU (the total scores of each variable were used as the observed variables). Figure 1 depicts the standardized solution for the structural model of the studied variables. The model was a saturated model: χ^2^ (0) = 0, RMSEA = 0.32, TLI = 1.00, CFI = 1.00. All the standardized path coefficients were presented in this model; the dotted lines were not significant, whereas the solid lines were significant. We discovered that online shopping (β = 0.28, *p* < 0.01), online pornography (β = 0.04, *p* < 0.01), SNS usage (β = 0.28, *p* < 0.01), and Internet gaming (β = 0.38, *p* < 0.01) had significant effects on GPIU. Additionally, we conducted a series of different tests to determine which was the most critical predictor of GPIU among online shopping, online pornography, SNS usage, and Internet gaming. According to Kim’s (1993) suggestion, the results showed that the regression coefficient of Internet gaming was bigger than the regression coefficients of online shopping (?χ^2^/?*df* = 183.17, *p* < 0.01), online pornography (?χ^2^/?*df* = 460.40, *p* < 0.01), and SNS usage (?χ^2^/?*df* = 57.93, *p* < 0.01), which suggested that Internet gaming was the most critical predictor of GPIU. Based on these findings, hypothesis (i) was verified.

### Descriptive Statistics and Difference Tests

MANOVA was used to determine gender differences in relation to online shopping, online pornography, SNS usage, Internet gaming, and GPIU in students from elementary school to university level. The results indicated that the gender × educational level interaction exerted a significant main effect on online shopping [*F*(3, 5204) = 4.35, *p* < 0.01]. Simple effect analysis demonstrated that female students had higher levels of online shopping than male students among junior school [*F*(2, 1707) = 14.17, *p* < 0.01], senior school [*F*(2, 685) = 7.04, *p* < 0.01], and university [*F*(2, 2,268) = 15.90, *p* < 0.01], while no difference was found in elementary school [*F*(2, 544) = 0.13, *p* > 0.05]. The gender × educational level interaction exerted a significant main effect on online pornography [*F*(3, 5,204) = 23.02, *p* < 0.01]. Simple effect analysis demonstrated that male students had higher levels of online pornography than female students among junior school [*F*(2, 1,707) = 9.69, *p* < 0.01], senior school [*F*(2, 685) = 11.80, *p* < 0.01], and university [*F*(2, 2,268) = 120.99, *p* < 0.01], while no difference was found in elementary school [*F*(2, 544) = 1.62, *p* > 0.05]. The gender × educational level interaction exerted a significant main effect on SNS usage [*F*(3, 5,204) = 3.89, *p* < 0.01]. Simple effect analysis demonstrated that female students had higher levels of SNS usage than male students in university [*F*(2, 2,268) = 8.83, *p* < 0.01], while no difference was found among elementary school [*F*(2, 544) = 0.50, *p* > 0.05], junior school [*F*(2, 1,707) = 1.12, *p* > 0.05], and senior school [*F*(2, 685) = 0.70, *p* > 0.05]. The gender × educational level interaction exerted a significant main effect on Internet gaming [*F*(3, 5,204) = 2.92, *p* < 0.05]. Simple effect analysis demonstrated that male students had higher levels of Internet gaming than female students among elementary school [*F*(2, 544) = 37.74, *p* < 0.01], junior school [*F*(2, 1,707) = 85.11, *p* < 0.01], senior school [*F*(2, 685) = 26.92, *p* < 0.01], and university [*F*(2, 2,268) = 173.84, *p* < 0.01]. The gender × educational level interaction exerted a significant main effect on GPIU [*F*(3, 5,204) = 11.45, *p* < 0.01]. Simple effect analysis demonstrated that male students had higher levels of GPIU than female students among elementary school [*F*(2, 544) = 14.96, *p* < 0.01], junior school [*F*(2, 1,707) = 10.20, *p* < 0.01], and university [*F*(2, 2,268) = 3.88, *p* < 0.01], while no difference was found in senior school [*F*(2, 685) = 0.36, *p* > 0.05]. Based on these results, hypothesis (ii) was verified.

## Discussion

The present study evaluated the association between SPIU and GPIU and found that the effect of Internet gaming on GPIU was bigger than the effects of online shopping, online pornography, and SNS usage, which was an important finding. Although the cognitive–behavioral model had distinguished the SPIU and GPIU, it did not distinguish the association between SPIU and GPIU. From this point of view, the results of the present study expanded the cognitive–behavioral model to some extent. Additionally, the results of the present study have found that gender differences in SPIU and GPIU were different across educational levels, which was also important for the development of a cognitive–behavioral model. Although some previous studies have found gender differences and educational level differences in SPIU and GPIU (e.g., Bianchi and Phillips, 2005; Baron and Campbell, 2012; Andreassen and Pallesen, 2014; Lepp et al., 2014; Griffiths, 2015; Griffiths et al., 2017; Piko et al., 2017), these studies did not evaluate gender differences across different educational levels. These two important findings were discussed with related theories, respectively.

### Association Between SPIU and GPIU

Based on the aforementioned findings, online shopping, online pornography, SNS usage, and Internet gaming were predictors of GPIU, which is consistent with previous studies (Lee and LaRose, 2007; Ballester-Arnal et al., 2016; Griffiths et al., 2016; Ding et al., 2017; Mcdaniel et al., 2017; Zhao et al., 2017; Tian et al., 2018). Crucially, in the present study, it was revealed that, after controlling for demographic variables, Internet gaming was the most vital predictor of students’ GPIU. It may be that Internet gaming can involve online shopping, SNSs, and online pornography. More specifically, there are different types of Internet gaming, such as social Internet gaming, violent Internet gaming, pornographic Internet gaming, and Internet gaming involving shopping; one Internet game even includes all the aforementioned functions. For example, a highly prevalent Internet game worldwide called League of Legends is characterized by intense, fast-paced competition between two teams of players (Kim et al., 2017). During the game, players need to communicate with each other to develop a strategy for attacking other teams; furthermore, they can buy equipment (such as weapons, skins, and blood bottles) for their characters with game currency through an Internet store (Kim et al., 2017). Based on these findings, Internet gaming is the most critical predictor of students’ GPIU compared with online shopping, online pornography, and SNSs.

However, Montag et al. (2015) investigated the association between GPIU and SPIU, and the results indicated that SNS usage (β = 0.68, *p* < 0.01) was the most critical predictor of GPIU rather than online shopping (β = 0.68, *p* < 0.29), online pornography (β = 0.42, *p* < 0.01), and Internet gaming (β = 0.45, *p* < 0.01). Several reasons might explain why their results were not consistent with our results. Firstly, a different sample was used. Students with a mean age of 16.2 years from different educational levels were followed in our study, whereas the mean age of the sample analyzed by Montag et al. was 19.98 years, and all of the participants were university students. The results of our study indicated that SNS usage tended to increase faster than Internet gaming, and the sample with only university students may have overstated the effect of students’ SNS usage by Montag et al. Secondly, in the study of Montag et al., multiple regression analyses rather than an SEM were used to examine the effects of online shopping, online pornography, SNS usage, and Internet gaming on GPIU. A SEM can control measurement errors from both independent and dependent variables, whereas multiple regression analysis can only control measurement errors from independent variables (Schuurman et al., 2016). Furthermore, an SEM can control the multicollinearity (all of the variance inflation factors of each variable were smaller than 5, which indicated that there were no serious multicollinearity problems) among online shopping, online pornography, SNS usage, and Internet gaming through partial correlations, whereas a multiple regression analysis cannot (Hu and Bentler, 1999). As stated in Section “Introduction,” online shopping, online pornography, SNS usage, and Internet gaming overlap with each other to a certain extent. These limitations in multiple regression analysis may have caused a measurement error for the subsequent analysis of the effects of online shopping, online pornography, SNS usage, and Internet gaming on GPIU. Thirdly, a different conception of Internet gaming and SNS usage was adopted. The frequency of engagement in online video games was employed to measure Internet gaming and Qzone was adopted as SNS usage in the study of Montag et al. In our study, Internet gaming was defined as a multidimensional structure that is related to continuation, preoccupation, negative consequences, escape, tolerance, loss of control, and giving up other activities because of excessive Internet gaming, and Qzone and WeChat usage was adopted to determine SNS usage. These different conceptions may have also led to different outcomes.

### Gender Differences in SPIU and GPIU for Students From Elementary School to University Level

This study was the first to evaluate gender differences in SPIU and GPIU in students from the elementary school level to the university level. The results indicated that from elementary school to university, gender differences in SPIU and GPIU tended to be changeable. More specifically, this could be as follows:

Firstly, for elementary school students, no gender differences were revealed in online shopping, online pornography, and SNS usage, whereas male students reported higher scores than female students in Internet gaming and GPIU. This is because, in elementary school, both male and female students rarely use the Internet to engage in online shopping, online pornography, and SNS usage due to being too young, whereas male students tended to use the Internet to engage in gaming-related activities for entertainment. It is possible that male students reported a higher score than female students in GPIU exclusively due to Internet gaming. In a previous study, intrinsic motivation was examined, which is related to the pleasure and satisfaction from engaging in a behavior, to explain the gender differences in Internet gaming. It is possible that males tended to have more experience with video games, which could help them overcome the challenges related to specific Internet games (Kober and Neuper, 2011). While males generally perceived more enjoyment than females during a complicated computer game, the intrinsic motivation of continuance behavior in relation to online gaming tended to increase (Wang and Wang, 2008). Furthermore, the evolved gender roles may be another factor that led to the gender difference in online gaming. For example, studies have suggested that females were more sensitive to frustrations in relation to online gaming than males (Liu and Alloy, 2010; Kober and Neuper, 2011); therefore, they may have perceived less enjoyment than males and, as a result, tended not to use the Internet to engage in gaming-related activities.

Secondly, in junior high school students, males reported higher scores than females in terms of online pornography, Internet gaming, and GPIU, whereas they scored lower in online shopping, and there were no gender differences in SNS usage. For online pornography, Ballester-Arnal et al. (2016) also reported these gender-related differences in online pornography in junior high school students (mean age = 14.76 years). This is because male students reported more arousal from online sexual activities than girls; therefore, male students were more likely to use the Internet to seek pornographic material for enjoyment (Shaughnessy et al., 2011; Ballester-Arnal et al., 2016). For online shopping, gender differences have been discussed, and the findings of Cho (2004) and Hasan (2010) indicated that more products associated with females, such as food, home wear, and clothing, were widely available online. The diversity of commercial products related to females may be a crucial factor leading to more female students shopping online than males. Furthermore, it has been reported that females enjoy the physical evaluation of products, such as seeing and feeling the product even though they may not necessarily buy them (Cho, 2004; Hasan, 2010), and female students may shop online solely for entertainment. Therefore, for junior high school students, online pornography and Internet gaming were the two critical predictors for male students’ GPIU, whereas online shopping was an important predictor for female students’ GPIU.

Thirdly, in senior high school students, males reported higher scores than females in online pornography and Internet gaming, but lower scores in online shopping, whereas there were no gender differences in SNS usage and GPIU. Similarly, for junior high school students, online pornography and Internet gaming were the two most crucial predictors for male students’ GPIU, whereas online shopping was the most critical predictor for female students’ GPIU. However, no gender differences were noted in GPIU. This may have been because female students’ engagement in online shopping increased faster with age than male students, which led to the gender differences in GPIU decreasing. Although male students’ viewing of online pornography increased faster with age than did female students’, the results of this study indicated that, after controlling for demographic variables, online shopping played a more crucial role than online pornography in predicting GPIU. The absence of an observed gender difference in GPIU may be due to the increase in online shopping for female students.

Finally, in university students, male participants reported higher scores than females in online pornography and Internet gaming, but lower scores in online shopping, SNS usage, and GPIU. Gender stereotypes can be used to interpret gender differences in the motivations for Internet use. More specifically, the perceived characteristics of female students are interdependence, a need to nurture, and a concern for others. They are more likely to use social media platforms such as Facebook, Qzone, and WeChat to obtain social or emotional support from others such as family members and peers who share similar experiences (Huan et al., 2014; Tian et al., 2015, 2017; Han et al., 2017). However, male students are typically perceived as independent, autonomous, and self-sufficient, and even though they seek less social or emotional support from others, they are more likely to use pornographic material or computer games for entertainment (Huan et al., 2014; Tian et al., 2017). Therefore, for university students, online pornography and Internet gaming were the two most critical predictors for male students’ GPIU, whereas online shopping and SNS usage were the most crucial predictors for female students’ GPIU. Additionally, female students reported higher scores than male students in GPIU. It may be that the effects of increased online shopping and SNS usage on female students’ GPIU were greater than that of increased online shopping, online pornography, and SNS usage on male students’ GPIU.

In summary, gender differences in online shopping, online pornography, SNS usage, Internet gaming, and GPIU were dynamic and changeable in students from the elementary school level to the university level. To interpret these results, it is necessary to explain inconsistent conclusions from other studies. Yang et al. (2014) examined GPIU in 1,173 junior high school students (mean age = 13 years), and the results suggested that male students reported higher levels of GPIU than did female students. In contrast, Jiang and Zhao (2017) investigated GPIU in 468 university students (mean age = 18.2 years), and the results indicated that female students reported higher levels of GPIU than did male students. These inconsistent conclusions can be explained by the results in our study that junior high school male students spent more time viewing online pornography and participating in Internet gaming for entertainment than did female students, whereas female university students engaged more in online shopping and SNS usage than did male students due to a desire to consume products and share their emotions.

## Implications for the Prevention of GPIU

According to our findings, some implications for the treatment and prevention of GPIU in school students are provided. Firstly, although, after controlling for demographic variables, online shopping, online pornography, SNS usage, and Internet gaming could positively predict GPIU, Internet gaming was the most important predictor of GPIU. Therefore, this finding suggests that interventions could be aimed at those participating in Internet gaming. Similarly, a previous study has suggested that education and training about the risks of excessive or compulsive Internet gaming can alleviate many symptoms of GPIU (Young and Case, 2004). Secondly, gender differences were revealed in online shopping, online pornography, SNS usage, Internet gaming, and GPIU for students from the elementary school level to the university level. This finding suggests that gender differences should be taken into consideration in interventions. For example, online pornography viewing tended to be unchangeable for female students, whereas the viewing of online pornography for male students tended to increase markedly from the elementary school level to the university level. Therefore, interventions related to online pornography should be aimed toward male students rather than female students. Finally, educational level differences were revealed in online shopping, online pornography, SNS usage, Internet gaming, and GPIU from elementary school students to university students. This finding suggests that educational level differences should be taken into consideration in interventions. For example, junior high school students had the highest scores in Internet gaming; therefore, interventions for Internet gaming should focus mostly on junior high school students.

## Limitations and Future Directions

Although the present study evaluated the effects of SPIU on GPIU and also examined the gender differences in these effects from elementary school to university level, there were also several limitations. Firstly, a cross-sectional design was used, which made it difficult to make a causal inference. Therefore, more future studies are needed to further evaluate the results of the present study. Secondly, the data of the present study have some skewness and kurtosis values that fall out of the accepted range, which may lead to analytical errors. Therefore, future studies should further verify the results of the present study with satisfactory skewness and kurtosis values. Finally, only Chinese students were evaluated in the present study, which made it difficult to generalize the results of the present study into other countries. More studies are needed to further verify the results of the present study, with samples from other countries.

## Data Availability Statement

The raw data supporting the conclusions of this article will be made available by the authors, without undue reservation.

## Ethics Statement

The present study was conducted in accordance with the 1964 Helsinki declaration and its later amendments or comparable ethical standards, with the approval of the Human Research Ethics Committee of Qingdao University of science and technology. Written informed consent to participate in this study was provided by the participants’ legal guardian/next of kin.

## Author Contributions

YT and TZ wrote the manuscript and data analysis. QS and LS conducted data analysis and interpretation of data for the manuscript. SC and NQ polished the manuscript and checked the manuscript.

## Conflict of Interest

The authors declare that the research was conducted in the absence of any commercial or financial relationships that could be construed as a potential conflict of interest.
